# Circulation of Dengue Virus Serotypes in the City of Makkah, Saudi Arabia, as Determined by Reverse Transcription Polymerase Chain Reaction

**DOI:** 10.1155/2017/1646701

**Published:** 2017-02-19

**Authors:** Sameer R. Organji, Hussein H. Abulreesh, Gamal E. H. Osman

**Affiliations:** ^1^Department of Biology, Faculty of Applied Science, Umm Al-Qura University, Makkah 21955, Saudi Arabia; ^2^Microbial Genetics Department, Agricultural Genetic Engineering Research Institute (AGERI), ARC, Giza 12619, Egypt

## Abstract

The present study was aimed to investigate the circulation of four dengue virus (DENV) serotypes in Makkah, Western Saudi Arabia. Blood samples were collected from 25 dengue fever-suspected patients and were subjected to molecular typing for DENV-1, DENV-2, DENV-3, and DENV-4 serotypes of dengue virus, by reverse transcription polymerase chain reaction (RT-PCR), using six sets of primers. Of the 25 samples, only six samples (24%) were found to be positive for dengue virus infection. The prevalence of DENV-1 was higher (50% of DENV-positive samples), as compared to DENV-2 (33.3%) and DENV-3 (16.6%) serotypes. The fourth serotype, DENV-4, was not detected in any of the DENV-positive samples. Although Makkah is considered endemic to dengue fever, we observed low prevalence of dengue virus in the city, which may be attributed to various factors. Nonetheless, the results presented herein confirm the circulation of DENV serotypes in the Western region of Saudi Arabia. To the best of our knowledge, the current study so far is the first report demonstrating the prevalence of the DENV-1 serotype in the city Makkah, Saudi Arabia.

## 1. Introduction

Dengue fever/dengue hemorrhagic fever (DF/DHF) is one of the major reemerging diseases in the past decade that has been expanding its circulation in various geographic locations around the world. The virus infection is characterized by a spectrum of illnesses that ranges from mild febrile illness to the fatal hemorrhagic disease and the dengue shock syndrome (DSS) [1–3]. Dengue virus infection constitutes a major public health concern and is estimated to cause about 300 million cases in a year across the globe. About 3.97 billion people, who are living in the epidemic areas in the tropical and subtropical regions, are at risk of acquiring the viral infection [2, 4].

Dengue fever/dengue hemorrhagic fever and the dengue shock syndrome are the diseases that are caused by dengue virus (DENV) that belongs to the genus* Flavivirus* a member of the Flaviviridae family. There are four dengue virus serotypes DENV-1, DENV-2, DENV-3, and DENV-4 that have similar antigenic properties but are distinct from one another in terms of specificity to the anti-DENV antibodies [2–4]. Dengue virus infection is an arthropod vector-borne disease that is transmitted by different species of* Aedes* mosquito (*Ae. aegypti*;* Ae. albopictus; Ae. polynesiensis*).* Ae. aegypti* is considered to be the main vector because it is in proximity to human habitation [1, 2, 4].

As reported by various epidemiological studies, the areas such as the South and Southeast Asia, South Africa, Africa, and the Arabian peninsula, which have a wide circulation of dengue viruses and/or their vectors, are considered to be dengue endemic areas [1, 2]. The dengue virus circulation was also reported in the regions that are located at the 35° N latitude (e.g., Korea and Japan), where the temperature in the winter season goes below 10°C at which the survival of* Ae. aegypti* becomes difficult [5, 6].

Saudi Arabia was considered to be a dengue-free country, until 1994, when the first dengue fever outbreak was reported in Jeddah that resulted in 289 confirmed cases of the viral infection [7]. The subsequent outbreaks occurred in different cities of the Western and Southwestern regions of Saudi Arabia, that is, Jeddah, Makkah, Aseer, and Jizan, in the years 1994 through 2010. By then the Western and Southwestern regions of Saudi Arabia were declared as the dengue endemic areas [8–13].

In the city of Makkah, the number of DF cases has increased since the first outbreak of the disease in 2004; however, only two reports are available, one published in 2008 [10] and the other published in 2009 [14], regarding the rise of DF cases and the prevalence of dengue virus serotypes in Makkah. As per the above-mentioned articles, DENV-2 and DENV-3 were the only serotypes detected in the cases of DF/DHF/DSS in Makkah [10]; however in one study by Shahin et al. [14], none of the DENV serotypes was identified. Thus, in such a dearth of information on the circulation of DENV serotypes in Makkah, we aimed to analyze the prevalence of DENV serotypes in DF-diagnosed patients in the city of Makkah, Western Saudi Arabia.

## 2. Methods

### 2.1. Sample Collection and Preparation

Blood samples (2.0 mL) from 25 patients presenting dengue-like clinical manifestations were collected in the commercially available EDTA anticoagulant tubes. The red blood cells were separated from the plasma by centrifugation at 3000 rpm for 5 min. The plasma was aspirated into a new tube and subjected to recentrifugation at 8000 rpm for 10 min. Most of the plasma was removed and approximately 100–200 *μ*L was left and stored at −70°C until further use. The blood samples were collected from four different hospitals in the city of Makkah.

### 2.2. Primers

The consensus primer pairs against dengue viruses D1 and D2 were designed using the Primer Explorer V4. The criteria for designing the serotype-specific primers (TS1, TS2, TS3, and DEN4) were based on the maximum homology of the primer pairs to the four DENV serotypes, high melting temperature, and nonhomology to the other regions of the dengue virus genome. All the oligonucleotides were commercially synthesized by Bioneer Corp., Korea, as described by Khawsak et al. [15] (Table 1).

### 2.3. RNA Extraction

The plasma samples of 25 suspected-case patients were subjected to RNA isolation by QIAamp viral RNA preparation kit (Qiagen, Heldin, Germany) as per the manufacturer's instructions; initially 560 *μ*L of the AVL buffer containing the carrier RNA was pipetted into a 1.5 mL microcentrifuge tube. A volume of 140 *μ*L of plasma sample was added to the buffer. The contents of the tube were mixed by pulse-vortex for 15 s and incubated at room temperature (25°C) for 10 min. The virus particles lysis was completed within 10 min at room temperature. The samples were briefly centrifuged to remove the drops from the inside of the lid. An aliquot of 560 *μ*L of absolute ethanol was added to the sample and mixed by pulse-vortex for 15 s. After mixing, the sample was centrifuged briefly to settle down the remaining drops in the inside of the lid. To ensure efficient binding, the samples were mixed thoroughly with ethanol to yield a homogeneous solution. Carefully, 630 *μ*L of the solution was applied to the QIAamp Mini column without wetting the rim, followed by centrifugation at 8000 rpm for 1 min. The QIAamp Mini column was placed into a clean 2.0 mL collection tube (provided with the kit), and the tube containing the filtrate was discarded. The samples with volumes greater than 140 *μ*L were repeated for this step until all of the lysate was loaded onto the spin column. A volume of 500 *μ*L of AW1 buffer was added to the column and centrifuged at 8000 rpm for 1 min. The QIAamp Mini column was placed into a clean 2.0 mL collection tube, and the tube containing the filtrate was discarded. An aliquot of 500 *μ*L of AW2 buffer was added to the spin column, and the column was centrifuged at full speed (14,000 rpm) for 3 min. The QIAamp Mini column was placed in a new 2.0 mL collection tube, and the old collection tube with the filtrate was discarded, followed by centrifugation at full speed for 1 min. The QIAamp Mini column was placed in a clean 1.5 mL microcentrifuge tube. The old collection tube containing the filtrate was discarded. The AVE buffer (60 *μ*L) was applied to the column. The cap was closed and incubated at room temperature for 3 min, followed by the last spin at 8000 rpm for 1 min for the elution of viral RNA.

### 2.4. Estimation of Quantity and Quality of Isolated RNA

The RNA concentrations of the 25 RNA samples that were isolated from 25 DF suspected patients were assessed using the NanoDrop® ND-1000 UV-Vis Spectrophotometer, which enables highly accurate analyses of extremely small samples with remarkable reproducibility. The sample retention system eliminates the need for cuvettes and capillaries, thereby allowing the estimation of viral RNA in a very low samples volume.

### 2.5. RT-PCR Amplification of Dengue Virus RNA (First Step)

Dengue virus RNA was first converted to a cDNA by the SuperScript III First-Strand Synthesis System (Invitrogen, Carlsbad, CA, USA) prior to the PCR. The cDNA was synthesized in a 0.2 mL RNAse-free microtube, starting with 5.0 *μ*L of viral RNA. The RNA samples were mixed with D1 and D2 synthetic oligonucleotide primers (0.5 *μ*L each), 1.0 *μ*L of 10 mM dNTP mix, and 3.0 *μ*L of DEPC-treated water, for a final volume of 10 *μ*L. This was incubated at 65°C for 5 min in the Applied Biosystems 96 Well Thermal Cycler to denature the RNA secondary structure and then placed on ice for 5 min. Next, 2.0 *μ*L 10x RT buffer (Invitrogen), 4.0 *μ*L of 25 mM MgCl_2_, 2.0 *μ*L 0.1 M DTT, 1.0 *μ*L RNase OUT (40 U/*μ*L), and 1.0 *μ*L Superscript III RT enzyme (200 U/*μ*L) were mixed in a separate RNAse-free 0.2 mL microtube and added to the RNA mixture on ice. This was incubated at 50°C for 50 min for cDNA synthesis and then at 85°C for 5 min to terminate the reactions in the Applied Biosystems 96 Well Thermal Cycler. The cDNA product was then chilled on ice for 5 min, and 1.0 *μ*L of RNase H was added to the tube and incubated at 37°C for 20 min to degrade the viral and carrier RNA. First-strand cDNA products were stored at −80°C until further use. PCR amplification was performed using primer pairs (forward dengue virus consensus primer D1 and reverse consensus primer D2), which was designed against the consensus cDNA sequence among the four dengue virus serotypes. The PCR product obtained from the RT-PCR amplification using D1 and D2 as primers was subsequently used as the template for PCR-based detection of four different DENV serotypes.

### 2.6. Multiplex PCR Amplification with Serotype-Specific Primers (Second Step)

The PCR amplification reaction was performed to identify the dengue virus serotype in the test samples. The reaction was conducted using the PCR products from the first step of the RT-PCR that used D1 and D2 as primers. A volume of 1.0 *μ*L of the diluted materials in the ratio of 1 : 100 (first RT-PCR solution to the Single Distilled H_2_O) was added to the PCR mixture containing all the components described for the amplification reaction using D1 as a forward and the dengue virus type-specific oligonucleotides (TS1, TS2, TS3, and DENV4) for each serotype as a reverse primer (Table 2). The PCR mixtures were subjected to 40 cycles (94°C for 30 s, 55°C for 60 s, and 72°C for 45 s) in a thermal cycler (Applied Biosystems). An aliquot of 15 *μ*L of the PCR reaction product was analyzed by electrophoresis (1.5% agarose gel in 1x TBE buffer). The size of the resulting bands was characterized for each dengue virus serotype with a 100 bp DNA ladder (GeneDireX, Taiwan).

## 3. Results

**Figure 1:**
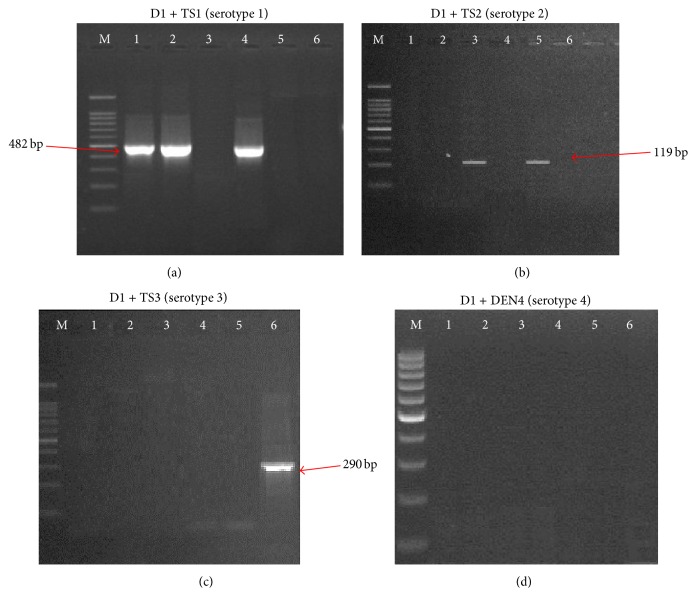
Identification of dengue virus serotypes by the specific primers. Four sets of specific primers were (a) D1 and TS1 for DENV-1, (b) D1 and TS2 for DENV-2, (c) D1 and TS3 for DENV-3, and (d) D1 and DEN4 for DENV-4.

The RT-PCR amplification of dengue virus RNA using D1 and D2 primer pair showed amplification of a 511 bp RNA fragment in the case of all the serotypes of dengue virus. The 511 bp RT-PCR product was used as a template for cDNA synthesis in the second step of RT-PCR, in which a single-tube multiplex PCR was performed using a set of serotype-specific primer pairs (D1, TS1, TS2, and DEN4). The primers used in the multiplex PCR showed highly specific amplification of viral RNA for each serotype with no nonspecific amplification of other non-DENV DNA, for example, human DNA target. Of the 25 blood samples analyzed in the present work, only six (24%) came out positive for DENV infection. The RT-PCR and a single-tube multiplex PCR were used to analyze the plasma specimens obtained from the 25 DF suspected patients in the city of Makkah, Western region of Saudi Arabia. Our results showed that, among the samples tested in this study, DENV-1 serotype was the most prevalent among the six DENV-positive samples (detected in three of six samples, 50%, Figure 1(a)). The occurrence of DENV-2 serotype (two of six samples, 33.3%, Figure 1(b)) was higher than DENV-3 serotype (one of six samples, 16.6%, Figure 1(c)) among the samples positive for dengue infection, whereas none of the samples demonstrated positivity for the DENV-4 serotype (Figure 1(d)).

## 4. Discussion

Dengue fever remains a serious health concern that has reemerged in the past ten years and affected thousands of individuals worldwide, especially those dwelling in the tropical and subtropical regions. Saudi Arabia, particularly the Western province, that is, Jeddah, Makkah, was declared as a DF-endemic area since the first outbreak in Jeddah in the year 1994. Our results continue to demonstrate the prevalence of dengue virus serotypes within the Western region of Saudi Arabia, particularly in the city of Makkah.

To the best of our knowledge, it is the first study identifying the highest prevalence of DENV-1 serotype in the patients with DF manifestations in Makkah city. In a study conducted in Jeddah, it was documented that the percentage of DENV-2 infection in DF-case patients is the highest compared to the DENV-1 or the DENV-3, with a dominance of the DENV-1 over the DENV-3 serotype [8]. However, in the present study, DENV-1 (50%) was found to be the most prevalent serotype that causes DF as compared to the DENV-2 (33.3%) or the DENV-3 (16.6%) serotypes. In a previous study by Khan et al. [10] higher prevalence of the DENV-2 serotype was reported in contrast to the DENV-3 in DF cases in Makkah, with no DENV-1 detection. DENV-1 appears to be the predominant dengue virus serotype in various regions, as it was the frequently detected DENV serotype in DF/DHF/DSS cases in the endemic areas of the Middle East, Asia, South America, or even in the nondengue endemic countries, such as Japan, where DENV is introduced by the travelers coming from the endemic regions [6, 16–18]. On the whole, it appears that DENV-3 has least circulation not only in the cases tested in current study, but also in the other patients belonging to the different areas in Saudi Arabia [7, 8, 10, 19] and elsewhere [20]. DENV-4 neither was detected in our study, nor has been detected in other investigations done so far on serological or molecular typing of dengue virus in Saudi Arabia [7, 8, 10, 19] or the other neighboring countries [20, 21]. Overall, DENV-1, DENV-2, and DENV-3 have been the predominant serotypes of dengue virus in the Middle East, particularly Saudi Arabia and Yemen [3], which is further confirmed by this study.

Although the incidence of dengue fever in Saudi Arabia has increased in the past three years (6512 cases in 2013; 2081 cases in 2014; 4312 cases in 2015) [22], the low prevalence of DENV detected in this study (24%) may be attributed, in part, to different factors. The total number of DF cases recorded in Saudi Arabia in the year 2015 was 4312, of which only 855 (19.8%) cases were recorded in Makkah, as compared to 3161 (73.3%) cases in Jeddah [22]. Most of these cases were recorded during the months from May through July, whereas in our study we analyzed the samples collected in the months other than the above. Overall, the prevalence of DENV in Saudi Arabia, as reported in different studies, ranged from 31.7% to 56.9% [13]. The other reason for such low prevalence of DENV infection in the present study might be the stage of disease or the day of sample collection after onset of the illness. It has been reported that the best period for DENV RNA detection in blood samples is in the acute phase of infection, that is, the first five days after onset of the symptoms [7], but, here, we did not know the stage of infection wherein the patients were sampled; thus, it is possible that those six positive samples were withdrawn from patients who were in the acute phase of infection. It is also worth noting that the negative samples might be of the patients who may not actually had DENV infection, in spite of presenting with dengue-like symptoms, the reason being that most of the hospitals in Saudi Arabia diagnose dengue virus infection by estimation of blood parameters, particularly, platelet counts which is only a predictive parameter in case of dengue fever [23].

## 5. Conclusion

Our results confirm the circulation of DENV-1, DENV-2, and DENV-3 in the city of Makkah, in the Western region of Saudi Arabia. The present work highlights the significance of continuous surveillance of DENV serotypes in Makkah, as annually during pilgrimage (Hajj) and the minor pilgrimage (Umrah), more than 3 million people visit the city from different geographic locations including areas that are DF-endemic zones. Novel serotypes, like DENV-4 and DENV-5, may emerge in the city of the entire region by the travelers (who wish to perform religious rituals) or the locals who travel to dengue endemic areas for vacation and holidays. Thus, it is highly imperative to discriminate the local origin serotypes from the traveler-introduced serotypes, for which continued surveillance is vital.

## Figures and Tables

**Table 1:** The oligonucleotide primers used in RT-PCR (first and second strand).

Primer	Primer sequence (5′ to 3′)
D1	TCAATATGCTGAAACGCGCGAGAAACCG
D2	TTGCACCAACAGTCAATGTCTTCAGGTTC
TS1	CGTCTCAGTGATCCGGGGG
TS2	CGCCACAAGGGCCATGAACAG
TS3	TAACATCATCATGAGACAGAGC
DEN4	TGTTGTCTTAAACAAGAGAGGTC

**Table 2:** Dengue virus specific primers and their expected product size.

Serotype	Primer pair	Size of PCR products (bp)
First step: RT-PCR		
All serotypes	D1 + D2	511
Second step: serotype detection		
DENV-1	D1 + TS1	482
DENV-2	D1 + TS2	119
DENV-3	D1 + TS3	290
DENV-4	D1 + DEN4	392
